# Candidate genetic variants and antidepressant-related fall risk in middle-aged and older adults

**DOI:** 10.1371/journal.pone.0266590

**Published:** 2022-04-14

**Authors:** A. C. Pronk, L. J. Seppala, K. Trajanoska, N. Stringa, B. van de Loo, L. C. P. G. M. de Groot, N. M. van Schoor, F. Koskeridis, G. Markozannes, E. Ntzani, A. G. Uitterlinden, F. Rivadeneira, B. H. Stricker, N. van der Velde

**Affiliations:** 1 Department of Internal Medicine, Section of Geriatric Medicine, Amsterdam Public Health Research Institute, Amsterdam UMC, University of Amsterdam, Amsterdam, the Netherlands; 2 Department of Internal Medicine, Erasmus University Medical Center, Rotterdam, the Netherlands; 3 Department of Epidemiology and Biostatistics, Amsterdam UMC, Vrije Universiteit Amsterdam, Amsterdam Public Health Research Institute, Amsterdam, the Netherlands; 4 Department of Human Nutrition and Health, Wageningen University, Wageningen, the Netherlands; 5 Department of Hygiene and Epidemiology, School of Medicine, University of Ioannina, Ioannina, Greece; 6 Department of Health Services, Policy and Practice, Center for Research Synthesis in Health, School of Public Health, Brown University, Providence, RI, United States of America; 7 Center for Evidence Synthesis in Health, Brown University School of Public Health, Providence, RI, United States of America; 8 Department of Epidemiology, Erasmus University Medical Center, Rotterdam, the Netherlands; Universitat Pompeu Fabra, SPAIN

## Abstract

**Background:**

Antidepressant use has been associated with increased fall risk. Antidepressant-related adverse drug reactions (e.g. orthostatic hypotension) depend partly on genetic variation. We hypothesized that candidate genetic polymorphisms are associated with fall risk in older antidepressant users.

**Methods:**

The association between antidepressant use and falls was cross-sectionally investigated in a cohort of Dutch older adults by logistic regression analyses. In case of significant interaction product term of antidepressant use and candidate polymorphism, the association between the variant genotype and fall risk was assessed within antidepressant users and the association between antidepressant use and fall risk was investigated stratified per genotype. Secondly, a look-up of the candidate genes was performed in an existing genome-wide association study on drug-related falls in antidepressant users within the UK Biobank. In antidepressant users, genetic associations for our candidate polymorphisms for fall history were investigated.

**Results:**

In antidepressant users(n = 566), for rs28371725 (CYP2D6*41) fall risk was decreased in TC/variant allele carriers compared to CC/non-variant allele carriers (OR = 0.45, 95% CI 0.26–0.80). Concerning rs1057910 (CYP2C9*3), fall risk was increased in CA/variant allele carriers compared to AA/non-variant allele carriers (OR = 1.95, 95% CI 1.17–3.27). Regarding, rs1045642 (*ABCB1*), fall risk was increased in AG/variant allele carriers compared to GG/non-variant allele carriers (OR = 1.69, 95% CI 1.07–2.69). Concerning the *ABCB1*-haplotype (rs1045642/rs1128503), fall risk was increased in AA-AA/variant allele carriers compared to GG-GG/non-variant allele carriers (OR = 1.86, 95% CI 1.05–3.29). In the UK Biobank, in antidepressant users(n = 34,000) T/variant-allele of rs28371725 (CYP2D*41) was associated with increased fall risk (OR = 1.06, 95% CI 1.01–1.12). G/non-variant-allele of rs4244285 (CY2C19*2) was associated with decreased risk (OR = 0.96, 95% CI 0.92–1.00).

**Conclusion:**

This is the first study showing that certain genetic variants modify antidepressant-related fall risk. The results were not always consistent across the studies and should be validated in a study with a prospective design. However, pharmacogenetics might have value in antidepressant (de)prescribing in falls prevention.

## Introduction

Falls are a major public health problem in older adults. Annually, one-third of the people over the age of 65 years fall at least once a year and half of this group is at increased risk for recurrent falls [1]. Ten percent of fall incidents result in serious injuries such as fractures, head injuries, or even death [2, 3]. The high prevalence and injury rate lead to substantial healthcare costs. Fall injuries are among the 20 most expensive medical conditions among community-dwelling older adults [4]. Furthermore, older adults often experience a decline in functional status and social activities after a fall and report a reduced quality of life up to 9 months after the injury [3, 5]. Multiple intrinsic and extrinsic risk factors are associated with falling, with prescribed medications being one of the most important contributors [6, 7]. Antidepressants and their subclasses have consistently been associated with increased fall risk [8]. Antidepressant use may affect fall risk through several underlying pathways, among important cardiovascular and neurological effects [9, 10]. In general, falls can be considered a common adverse drug reaction (ADR) of antidepressant use.

Furthermore, ADRs are common in antidepressant users. The occurrence of ADRs other than falls and therapy effectiveness have been shown to be partly dependent on individual characteristics such as genetic variation [11–14]. Pharmacokinetic data underlines the significance of the cytochrome P450 (CYP) family in antidepressant metabolism. Especially, *CYP2D6* and *CYP2C19* are of major importance in the metabolization of many commonly prescribed antidepressants [11, 12, 15]. Functional genetic variants within these genes result in significant variation in enzymatic activity and subsequently, altered phenotypes (e.g. poor metabolism) and complementary pharmacokinetic parameters (e.g. higher drug concentration) [11, 14, 16]. Several other genes outside the CYP450 system have also been associated with antidepressants pharmacokinetics. For instance, the *ABCB1* gene encodes for P-glycoprotein (P-gp), a transmembrane protein located at the luminal membrane of the endothelial cells that form the blood-brain-barrier (BBB). At the BBB, P-gp acts as an active efflux pump and regulates intracerebral concentrations and may affect the clinical response of drugs that are substrates for this transporter, such as antidepressants. Genetic variants within *ABCB1* have been associated with variation of treatment response of antidepressant therapy and conflicting results exist with respect to adverse events [17–19].

Different clinical prescribing guidelines for antidepressants exist (e.g. the Clinical Pharmacogenetics Implementation Consortium (CPIC) and the Dutch Pharmacogenetic Working Group (DPWG)), providing recommendations regarding dosing and drug choice based on predicted enzymatic activity [20–22]. Genotype based dose adjustments might be of value in reducing the risk of medication-related falls since the risk for falls has been shown to be dose-dependent [23–25]. In this case, falls prevention could be one of the target areas for pharmacogenetic testing. Therefore, our aim was to assess if candidate polymorphisms are associated with fall risk in antidepressant users.

## Methods

### Identification of genetic polymorphisms

The Pharmacogenomics Knowledgebase was used to identify relevant single nucleotide polymorphisms (SNPs) for the pharmacokinetics and adverse effects of antidepressants [26]. For supporting literature, a structured explorative search in PubMed was conducted by the first author (June 2019). The MESH-terms “antidepressants agents”, “selective serotonin reuptake inhibitors”, “tricyclic antidepressive agents”, “serotonin and noradrenaline reuptake inhibitors”, “pharmacogenetics” or “single nucleotide polymorphism” and “pharmacokinetics” or “adverse events” were combined in the search. We searched for clinically relevant SNPs for commonly prescribed antidepressants: paroxetine, (es)citalopram, sertraline, fluoxetine, amitriptyline, nortriptyline, clomipramine, imipramine, duloxetine, mirtazapine and venlafaxine. The following non-rare variants (minor allele frequency (MAF) >1% in Caucasian population) that could be genotyped from genome-wide arrays were identified [27, 28]: CYP1A2: rs762551 (*1F); CYP3A4: rs35599367 (*22); CYP3A5: rs776746 (*3); CYP2C9:rs1799853 (*2); rs1057910 (*3); CYP2D6: rs28371725 (*41); rs3892097 (*4); CYP2C19: rs4244285 (*2), rs12248560 (*17); ABCB1: rs1045642, rs1128503. Chromosome positions for these different SNPs are shown in S1 Table.

### Study population

Firstly, we investigated whether candidate genetic polymorphisms modify the association between antidepressant use and fall risk in a harmonized cohort of Dutch studies. Subsequently, we assessed if these polymorphisms were associated with fall risk in an existing genome-wide association study (GWAS) for drug-related falls conducted in UK Biobank (UKBB) Study.

For the candidate gene analysis, data from an existing cohort created for AD*F*ICE_IT study was used [29]. This is a harmonized cohort of five European cohorts (the Longitudinal Aging Study Amsterdam (LASA), the B-vitamins for the Prevention Of Osteoporotic Fractures (B-PROOF), the Rotterdam Study (or Erasmus Rotterdam Gezondheid Onderzoek (ERGO), the Irish Longitudinal Study on Ageing and the Activity and Function in the Elderly in Ulm study). Since only the LASA, B-PROOF and ERGO cohort have genetic data available, this subset was included in the current study. More detailed description of these cohorts can be found in S1 Appendix. Subsequently, we leveraged data from a genome-wide association study (GWAS) for drug-related falls performed in UKBB.

All studies were approved by medical ethics committees. For the B-PROOF study the Medical Ethics Committee (METC) of Wageningen University approved the study protocol [30]. The LASA study received approval by the METC of the VU University Medical Center [31]. The Rotterdam study received approval by the METC of the Erasmus MC and the Dutch Ministry of Health, Welfare and Sport [32]. The UKBB has approval from the North West Multicentre research ethics committee (MREC) as a research Tissue Bank (RTB) approval. This approval means that researchers do not require separate ethical clearance and can operate under the RTB approval [33, 34]. In all studies, all participants gave written informed consent before entering the study. All experimental studies were conducted according to principles in the Declaration of Helsinki.

### Falls

Fall occurrence during the past 12 months was used as outcome measure. In the harmonized cohort, the occurrence of falls in the past 12 months was based on retrospective reporting by questionnaires in person or through a telephone contact. In the UKBB, self-reported data about falls was gathered through a touch screen questionnaire.

### Antidepressant use

Anatomical Therapeutic Chemical (ATC) codes were used to define antidepressant use in all cohorts. Our exposure of interest was antidepressant use (N06A and N06CA). In addition, we defined non-selective monoamine reuptake inhibitor (N06AA, N06CA01, and N06CA02), selective serotonin reuptake inhibitor (N06AB and N06CA03) and other antidepressant (N06AF, N06AG and N06AX) use. N06AF, N06AG categories were combined with N06AX (into category: ‘other antidepressants’) as there needed to be sufficient amount of users per variable. The antidepressant variables needed to be used by >1% of the participants.

In the LASA cohort, use of medication was assessed from questionnaires and checked with medication boxes provided by participants [35]. In B-PROOF, self-reported medication use was recorded by questionnaires at baseline [30]. In the Rotterdam study, medication use was registered during the home interview by participants presenting all the medication they were using at the day of the interview [36].

In the UKBB, self-reported regular medication and health supplements taken weekly, monthly or every three months were recorded at baseline [37]. Medication and health supplements data (Data field:20003) were manually mapped to identify antidepressant users.

### Covariates

In the harmonized cohort, characteristics including age, gender, living situation, educational level, pain, smoking habits and alcohol use were assessed using a questionnaire. During study visits, various characteristics were measured, including weight, height, blood pressure, depressive symptoms, anxiety symptoms and cognitive status (Mini-mental state examination (MMSE)). Additionally, serum creatinine levels were determined. Hand grip strength, balance and gait speed were assessed as measures of physical function. A detailed description of the covariate assessment in the harmonized cohort and the harmonization procedure is presented in S2 Appendix & S2 Table. Possible mediators between antidepressant use and falls (dizziness, balance, hypotension, gait speed) were not tested as covariates.

### Genotyping

Genotyping for the first LASA cohort (participants at the C wave) was performed by two arrays Axiom-NL Array and the Infinium Global Screening Array (GSA) [38]. The latter was also used for the genotyping of participants in 3B wave. Furthermore, Illumina-Omni express array was used in B-PROOF, and genotyping in the Rotterdam Study, was carried out with the Illumina 550, 550 duo and Human 610 Quad arrays [30, 39]. After initial genotyping, stringent quality control was performed in all cohorts. The imputation data was based on the Haplotype Reference Consortium (HRC) panel version 1.1 in these cohorts, and the process was performed on the Michigan Imputation server. The imputation quality was good with an R^2^ value higher than 0.88, 0.87 and 0.81 for the B-PROOF, LASA and Rotterdam Study, respectively. To extract the hard genotype calls (0,1 or 2) of relevant SNPs, PLINK version 1.90 was used [40]. In addition, the variants within *CYP2D6*, *ABCB1* and *CYP2C9* were combined into composed metabolism phenotype (S3 Table).

The majority of UKBB participants were genotyped with the Affymetrix UK Biobank Axiom Array (Santa Clara, CA, USA), while 10% of participants were genotyped with the Affymetrix UK BiLEVE Axiom Array. Detailed quality control and imputation procedures are described elsewhere [41]. Imputation was performed using the HRC Panel. When a SNP was not present, then the UK10K and 1000 Genomes phase 3 reference panels were used [42, 43].

### Statistical analysis

In the harmonized cohort, baseline characteristics were determined for antidepressant users and non-antidepressant users. Differences between groups were tested using a t-test for normally distributed variables, Mann-Whitney U test for non-normally distributed continuous variables and a Chi-square test for categorical variables. Deviation of allele frequencies from Hardy-Weinberg equilibrium (HWE) was assessed using a Chi-Square test.

To investigate the association between antidepressant use (and subgroups of antidepressants) and falls, logistic regression models were conducted. Initially, the models were adjusted for age and gender (model 1). Additionally, a second model (model 2) was examined assessing the following variables as potential covariates: cohort index, age, gender, education, body-mass index, alcohol use, smoking habits, use of a walking aid, living situation, MMSE, depressive symptoms, anxiety symptoms, diabetes, grip strength, co-medication, pain and kidney function. The covariates were examined separately and included in the model if they changed the Odds Ratio (OR) of the association between antidepressant use and falls by at least 10%.

To account for missing covariates, missing data was imputed in five copies with imputations by chained equation and the parameter estimates were pooled using Rubin’s rules [44]. Among the 27 variables that were included in this analysis, there were 13 variables without missing values. The median of the proportion of missing values for the 14 variables with missing values was 8.9% (interquartile range, 0.6%-13.1%). We also tested the correlation between antidepressant use and the variables included in model 2 by Spearman’s correlation coefficient. Various cut-offs (0.4–0.85) have been applied in the literature [45]. We applied the more restrictive cut-off of ≥0.4 for the coefficient to be sure that the multicollinearity problem is not present in our analyses.

To test for effect modification, an interaction term between the medication group and the SNP or composed metabolism phenotype was added to the age- and gender adjusted model (model 1). The exposure variable was first restrained to antidepressants relevant to the specific SNP (substrate specific) or metabolism phenotype according to the literature. Subsequently, the exposure variable was expanded to all antidepressants to create more power. In case of a p-value of the interaction term <0.1, further analyses for this SNP were conducted. The association between genotype and fall risk was assessed in antidepressant users. In addition, fall risk for antidepressants users was compared to non-users, stratified for different genotype carriers based on the individual SNPs and composed metabolism phenotypes.

Secondly, candidate gene analyses were performed in data from a GWAS for drug-related falls performed in UKBB. Users of 1) all antidepressants, 2) Tricyclic antidepressants (TCAs), 3) Selective Serotonin Reuptake Inhibitors (SSRIs) were selected and genetic association analyses for falls were performed in these subgroups. Logistic regression models were used and adjusted for age, gender and the first 15 principal components. Associations between SNPs and fall risk were tested for all autosomal variants. Individuals were excluded based on unusually high heterozygosity or >5% missing genotype rate and on a mismatch between self-reported and genetically-inferred sex. SNP exclusions were made based on low MAF (<1%), HWE (p >1 x 10^−06^) and SNP call rate (>95%). We investigated the results of these analyses for the candidate SNPs mentioned above. P-values less than 0.05 were considered statistically significant.

Statistical analyses were conducted in SPSS (version 25.0, IBM, Armonk, NY, USA) and in R (version 3.6.1) using packages mice and miceadds.

## Results

### Harmonized cohort

#### Study population

Fall and medication data was available for 11,765 participants. Of them, 26.6% reported a fall in the past 12 months. In total, for 9,335 participants genetic data was available. In this group, 26.5% of the participants experienced a fall in the past 12 months. The baseline characteristics of antidepressant users and non-antidepressant users are shown in S4 Table. In summary, antidepressant users were more often female, more often smokers, experienced more anxiety and depressive symptoms and reported more often dizziness and pain. Antidepressant users had poorer physical function parameters and experienced more often hypotension compared to non-antidepressant-users. Also, antidepressant users used more medication including more often benzodiazepines and opioids.

#### Antidepressant use and fall risk

Of the 11,765 participants, 700 participants (5.9%) were antidepressant users. Antidepressant use was associated with increased fall risk in the fully adjusted model (OR 1.58, 95% CI 1.34–1.87) and similarly to subclasses of antidepressants. The results are shown in Table 1. There were no signs of significant multicollinearity between the variables included in model 2 and antidepressant use.

**Table 1:** Association between antidepressant use and fall occurrence in past 12 months.

	N	Model 1 (OR 95% CI) ^a^	Model 2 (OR 95% CI) ^b^
**Antidepressant use**	700	1.81 (1.54–2.13)*	1.58 (1.34–1.87)*
**Classified by type of antidepressant**			
SSRI	376	1.86 (1.77–2.19)*	1.53 (1.23–1.90)*
TCA	199	1.72 (1.29–2.31)*	1.43 (1.07–1.92)*
Other Antidepressants	138	1.88 (1.33–2.67)*	1.51 (1.06–2.15) *

Data is presented in odds ratio and 95% confidence interval; N = Number of antidepressant users from 11,765 participants included in analysis. SSRI = Selective Serotonin Reuptake Inhibitor; TCA = Tricyclic Antidepressants.

a Model 1 was adjusted for age and gender.

b Model 2 of antidepressant and SSRI use was adjusted for age, gender and depressive symptoms. Model 2 of TCA and other antidepressant use was adjusted for age, gender, depressive symptoms and number of used medications.

* statistically significant at p<0.05.

#### Genotypes and antidepressant-related fall risk

Allele frequencies of the analyzed genotypes and the chi-square tests of the distribution according to HWE are presented in S5 Table. Allele frequencies of rs1799853 (CYP2C9*2) deviated significantly from HWE. Sensitivity analyses in each cohort showed a significant deviation only in the LASA C Cohort (S6 Table).

When the exposure term was limited to antidepressants being relevant for the metabolism of the specific SNP, interaction term including rs28371725 (CYP2D6*41) was significant. When the exposure term was ‘all antidepressants’, significant interaction terms were found for the following SNPs: rs28371725 (CYP2D6*41), rs28371725/rs3892097 (CYP2D6*41/CYP2D6*4) combined into *CYP2D6* phenotype, rs1057910 (CYP2C9*3), rs1045642 (*ABCB1*) and rs1045642/rs1128503 combined into *ABCB1* haplotype.

The results of the associations between *CYP2D6* genotype and fall risk within antidepressant users are presented in Table 2. In antidepressant users, fall risk was decreased in the heterozygote TC (variant) allele carriers of rs28371725 (*41) compared to CC (non-variant) allele carriers (OR 0.45, 95% CI 0.26–0.80). Also, carriers of at least one variant allele (TC and TT) of rs28371725 had significant decreased fall risk compared to non-variant allele (CC) carriers (OR 0.47, 95% CI 0.27–0.80). When *4 and *41 were combined into *CYP2D6* phenotypes, no significant associations were found. The antidepressant users in different CYP2D6*41 genotype carriers and for the CYP2D6 phenotype genotype carriers did not differ in terms of Fall risk increasing drug (FRID) use, CYP2D6 inhibitor or CYP2D6 inducer use.

**Table 2:** Association between CYP2D6*41 and CYP2D6*41/*4 genotype and fall risk within antidepressant users.

	All antidepressant users			Substrate specific antidepressant users ^#^		
CYP2D6 *41	N	Model 1	P-value	N	Model 1	P-value
CC	484	Ref		304	Ref	
TC	75	0.45 (0.26–0.80)	0.006*	49	0.47 (0.23–0.94)	0.034*
TT	7	0.61 (0.12–3.22)	0.559	4	-	-
						
CC	484	Ref.		304	Ref.	
Variant allele carriers (TC and TT)	82	0.47 (0.27–0.80)	0.006*	53	0.43 (0.21–0.85)	0.016*
**CYP2D6 phenotype**	**N** ^**#**^	**Model 1**	**P-value**	**N** ^**#**^	**Model 1**	**P-value**
EM	516	Ref		326	Ref	
IM	19	0.30 (0.08–1.04)	0.058	12	0.32 (0.07–1.50)	0.147
PM	31	1.38 (0.66–2.90)	0.398	19	1.39 (0.54–3.59)	0.494
						
EM	516	Ref		326	Ref.	
IM and PM	50	0.84 (0.45–1.57)	0.589	31	0.86 (0.39–1.88)	0.704

Data is presented in odds ratio and 95% confidence interval.

N = number of participants per genotype. EM = Extensive metabolizer; IM = Intermediate metabolizer; PM = Poor metabolizer; Model 1 was adjusted for age and gender.

^#^ users of the following antidepressants were included in the analysis: *amitriptyline*, *nortriptyline*, *clomipramine*, *imipramine*, *paroxetine*, *fluvoxamine*, *citalopram*, *sertraline*, *doxepin*, *duloxetine*, *mirtazapine*, *venlafaxine*, *trazodone*.

*statistically significant at p<0.05.

The results of the associations between antidepressant use and fall risk stratified for the genotypes of *CYP2D6* are presented in S7 Table. Antidepressant use was associated with increased fall risk in the non-variant allele (CC) carriers of rs28371725 (*41) (OR 1.86, 95% CI 1.53–2.26). No significant association was found in the category of at least one variant allele carriers (TC and TT). When *4 and *41 were combined into phenotypes, antidepressant use was associated with increased fall risk in the extensive metabolizer (EM) (OR 1.73, 95% CI 1.43–2.09) and in the poor metabolizer (PM) phenotype (OR 2.17, 95% CI 1.02–4.61).

The results of the associations between *CYP2C9* genotype and fall risk within antidepressant users are presented in Table 3. An increased fall risk was found in the heterozygote allele (CA) carriers of rs1057910 compared to non-variant allele carriers (AA) (OR 1.95, 95% CI 1.17–3.27). The antidepressant users in different CYP2C9*3 genotype carriers did not differ in terms of FRIDs use, CYP2C9 inhibitor or CYP2C9 inducer use.

**Table 3:** Association between CYP2C9*3 and fall risk within antidepressant users.

	All antidepressant users			Substrate specific antidepressant users ^#^		
CYP2C9 *3	N	Model 1	P-value	N	Model 1	P-value
AA	496	Ref		113	Ref	
CA	70	1.95 (1.17–3.27)	0.011*	17	1.79 (0.62–5.16)	0.283
CC	-	-		-		
Any variant allele carriers (CA & CC)	70	1.95 (1.17–3.27)	0.011*	17	1.79 (0.62–5.16)	0.283

Data is presented in odds ratio and 95% confidence interval.

N = number of participants per genotype Model 1 was adjusted for age and gender.

^#^ users of the following antidepressants were included in the analysis: *fluoxetine (and psycholeptics)*,*escitalopram*, *amitriptyline (and psycholeptics)*.

*statistically significant at p<0.05.

The results of the associations between antidepressant use and fall risk stratified for the genotypes of rs1057910 of the *CYP2C9* gene are presented in S8 Table. Antidepressant use was associated with increased fall risk in the non-variant allele (AA) carriers (OR 1.56, 95% CI 1.28–1.89), in the heterozygote (CA) allele carriers (OR 2.98, 95% CI 1.82–4.88) and in the carriers of at least one variant allele (CA or CC) (OR 3.02, 95% CI 1.84–4.93).

The results of the associations between rs1045642 genotype and rs1045642/rs1128503 haplotype genotype and fall risk in antidepressant users are presented in Table 4. An increased fall risk was found in the heterozygote variant allele (AG)-carriers compared to non-variant (GG) allele carriers (OR 1.69, 95% CI 1.07–2.69) as well as in GA and AA variant allele carriers compared to non-variant allele (GG) carriers (OR 1.65, 95% CI 1.06–2.55). Regarding the haplotype, variant allele (AAAA) carriers had an increased fall risk compared to non-variant allele (GGGG) carriers (OR 1.86, 95% CI 1.05–3.29) as well as the combined variant allele (AAAA or GAGA)-carriers (OR 1.72, 95% CI 1.07–2.77). The antidepressant users in different ABCB1 genotype carriers did not differ in terms of FRIDs use, P-gp inhibitor or P-gp inducer use.

**Table 4:** Association between ABCB1 rs1045642 and rs1045642/rs1128503 haplotype and fall risk within antidepressant users.

	All antidepressant users		
rs1045642	N	Model 1	P-value
GG	127	Ref.	
GA	271	1.69 (1.07–2.69)	0.025*
AA	168	1.57 (0.95–2.60)	0.080
Variant allele carriers (GA and AA)	439	1.65 (1.06–2.55)	0.025*
**rs1045642/rs11285003**			
GGGG	115	Ref.	
GAGA	190	1.65 (0.99–2.74)	0.054
AAAA	103	1.86 (1.05–3.29)	0.034*
Variant allele carriers (GAGA & AAAA)	293	1.72 (1.07–2.77)	0.026*

Data is presented in odds ratio and 95% confidence interval. N = number of participants per genotype. Model 1 was adjusted for age and gender.

*statistically significant at p<0.05.

The results of the associations between antidepressant use and fall risk stratified for the genotypes of rs1045642 and rs1045642/rs1128503 haplotype of the *ABCB1* gene are presented in S9 Table. Antidepressant use was associated with an increased fall risk in the heterozygote variant allele (AG)-carriers (OR 1.92, 95% CI 1.48–2.48), in the homozygote variant allele (AA)-carriers (OR 1.83, 95% CI 1.31–2.54) and in AG or AA–carriers (OR 1.89, 95% CI 1.54–2.31). Regarding the haplotype, antidepressant use was associated with an increased fall risk in the heterozygote variant allele (GAGA)-carriers (OR 1.79, 95% CI 1.32–2.44), the homozygote variant allele (AAAA)-carriers (OR 2.19, 95% CI 1.44–3.33) and in the combined variant allele carriers (GAGA and AAAA) group (OR 1.92, 95% CI 1.50–2.46).

### UK Biobank

UKBB GWAS for drug-related falls included data from approximately 34,000 antidepressant users, almost 12,000 TCA users, and approximately 12,000 SSRI users. Around 35% of the antidepressant users reported at least one fall in the past year. Table 5 shows the results of the association analysis for falls in antidepressant users. In antidepressant uses, T (variant) allele of rs28371725 located in *CYP2D6* gene (*41) was associated with increased risk of falling (OR 1.06, 95% CI 1.01–1.12). Furthermore, the G (non-variant) allele of rs4244285 located in *CYP2C19* gene (*2) was associated with decreased risk of falling (OR 0.96, 95% CI 0.92–1.00). S10 Table shows the results of the association analysis for SSRI users. In SSRI users, C (non-variant)-allele of rs1799853 located in *CYP2C9* gene (*2) was associated with increased risk of falling (OR 1.08, 95% CI 1.01–1.15). S11 Table provides the results for the TCA users. In TCA users, T (variant) allele of rs28371725 located in *CYP2D6* gene (*41) was associated with increased risk of falling (OR 1.12, 95% CI 1.02–1.22).

**Table 5:** Association between candidate SNPs and fall risk in antidepressant users in UKBB.

Gene	SNP	EA^a^	NEA^b^	EAF^c^	OR (95% CI)	P-value
ABCB1	rs1045642	A	G	0.54	1.01 (0.98–1.05)	0.448
	rs1128503	A	G	0.44	1.01 (0.98–1.05)	0.443
CYP3A4	rs35599367 (*22)	G	A	0.95	1.01 (0.94–1.09)	0.761
CYP3A5	rs776746 (*3)	C	T	0.93	1.00 (0.94–1.07)	0.945
CYP2C9	rs1057910 (*3)	A	C	0.94	0.99 (0.93–1.06)	0.854
	rs1799853 (*2)	C	T	0.86	1.02 (0.97–1.07)	0.416
CYP2C19	rs4244285(*2)	G ^d^	A ^e^	0.85	0.96 (0.92–1.00)	0.049*
	rs12248560 (*17)	C	T	0.79	1.00 (0.96–1.04)	0.982
CYP1A2	rs762551(*1F)	C	A	0.27	NA	NA
CYP2D6	rs28371725(*41)	T ^e^	C ^d^	0.10	1.06 (1.01–1.12)	0.026*
	rs3892097 (*4)	T	C	0.20	0.99 (0.95–1.03)	0.496

^a^ EA = Effect allele

^b^ NE = Non-effect allele

^c^ EAF = Effect allele frequency

^d^ normal function allele

^e^ deficient function allele.

*statistically significant at p<0.05.

## Discussion

We addressed the effect of genetic variation on antidepressant-related falls and thus the potential for applying pharmacogenetics in this clinically very relevant health problem. In line with our expectations, antidepressant use was associated with increased fall risk, and the association was significantly modified by SNPs mapping to *CYP2D6*, *CYP2C9*, *CYP2C19* and *ABCB1*. However, the findings were not always consistent across the studies.

To our knowledge, this is the first study that investigated the influence of pharmacogenetics in relation to antidepressant-related fall risk. We assessed this topic in two study populations, in the UKBB with a very big sample size of middle-aged to older antidepressant users and in the smaller harmonized cohort of Dutch studies of older participants, with thus an overall higher risk of antidepressant-related falls given the older age. To date, data on pharmacokinetic differences is scarce especially for the population of older adults.

For the variant alleles *4 and *41 of the *CYP2D6* gene, we hypothesized an increased fall risk due to absent or diminished antidepressant metabolism. *4 is the most common variant allele of *CYP2D6* with a MAF of 15.5% in Caucasians and the most frequent non-functional allele in poor metabolizers (PMs) [27, 46]. Due to absent *CYP2D6*-mediated metabolism, PMs have higher antidepressant plasma concentrations than EMs (normal enzymatic function) and are therefore more likely to suffer from dose-dependent ADRs [47, 48]. *41 is the most common deficient allele in Caucasians, being present among 3% and leading to decreased enzyme activity (creating the IM phenotype) [27]. Results from the UKBB population are partly in line with our hypothesis, since the *41 variant allele was associated with an increased fall risk (OR 1.06) but concerning *4 variant allele, no significant association was found. In the harmonized cohort for the *4 variant allele no association was found and in antidepressant users, fall risk was decreased in the heterozygote carriers of *41 compared to non-variant carriers. When *4 and *41 were combined into *CYP2D6* phenotype in the harmonized cohort, no significant associations were found between genotype and fall risk in antidepressant users. However, the number of participants in the IM and PM groups was low. These contradictory findings regarding *41 could be related to several factors. First, *CYP2D6* plays an important role in the metabolism of TCAs and the proportion of TCA users was higher in UKBB than in the harmonized cohort. Second, in the harmonized cohort the number of variant allele carriers was limited and this could have played a role in the conflicting results. Third, with increasing age, a possible decline in CYP-enzyme activity is described and the enzymatic differences due to genotype differences may become smaller with age. However, others state that there are no important age-dependent changes in CYP-enzyme activity (especially in *CYP2D6* activity) [24, 49].

Concerning *CYP2C19* *2 variant allele, we hypothesized an increased fall risk due to diminished/absent antidepressant metabolism in carriers of *2 variant. This was indeed observed in the UKBB. Since not-carrying *2 variant was associated with decreased fall risk, so carrying the *2 variant was associated with increased fall risk. *2 variant allele is the most common non-function allele in CYP2C19, with a MAF of 18.3% in the Caucasian population [20, 22, 27]. (Es)citalopram is being primarily metabolized by *CYP2C19* and differences in pharmacokinetic properties have been reported based on phenotype [50]. Furthermore, Fabbri et al. showed that PMs had a higher risk of adverse effects early in treatment, but also had better symptom improvement and remission probability than EMs during (es)citalopram treatment [51]. Moreover, *CYP2C19* is involved in metabolizing the tertiary amines of TCAs (e.g. amitriptyline) to secondary amines (e.g.nortriptyline) and patients may have more risk of treatment failure or adverse effects based on their genotype [21]. In our harmonized cohort, no association with fall risk was found for this variant allele, which is probably related to the small cohort size. Considering the modest effect found in UKBB, we do not expect there is sufficient power present in the harmonized cohort.

Furthermore, in UKBB among SSRI users, not-carrying *2 allele of *CYP2C9* was associated with increased fall risk, so carrying *2 allele is associated with decreased fall risk. *3 allele variant showed no association in UKBB. In our harmonized cohort, no significant association was found for *2 and significantly increased fall risk was found in the heterozygote and variant allele carriers of *3 compared to normal carriers. Both *2 and *3 variant alleles are the most common variants of *CYP2C9* and result in decreased or absent enzymatic activity. In Caucasians, *2 and *3 have MAF of about 12 and 8%, but result in poor metabolizer phenotype only in about 0.7% of Caucasians [15, 22, 52]. *CYP2C9* has been less studied in the field of antidepressant pharmacogenetics, since it does not play a primary role in the metabolism of antidepressants, but is involved in the secondary pathways [12]. *CYP2C9* forms the only secondary pathway of metabolism of fluoxetine, and therefore decreased enzymatic activity of *CYP2C9* could be of importance in co-existence with *CYP2D6* PM phenotype in fluoxetine users [12, 52]. *CYP2C9* genotypes should probably be investigated in conjunction with other metabolizing enzymes and considering the limited evidence related to the role of *CYP2C9* in antidepressant metabolism our results need to be replicated.

*ABCB1* is another candidate gene that might modulate antidepressant response [49]. In the harmonized cohort, in antidepressant users, fall risk was increased in the heterozygote (AG) genotype and variant allele carriers of the rs1045642 SNP compared to the GG (non-variant) genotype carriers. In the UKBB population, no association with fall risk was found. Polymorphisms of the *ABCB1* gene appear to be correlated to expression levels and function of P-gp. These SNPs have been associated with changes in pharmacokinetics of substrate drugs, treatment response and adverse effects [53]. However, most studies that analyzed the relation of *ABCB1* gene variants with antidepressants have focused on treatment response and show contradictory results [18, 19, 54–56]. A significant association between rs1045642 and the risk of postural hypotension in nortriptyline users has been shown [18]. Noteworthy is the importance of postural hypotension as a fall risk factor. Furthermore, the *ABCB1* gene is large and highly polymorphic. Rs1045642(3435C>T) shows strong linkage disequilibrium with rs1128503(1236C>T) and rs2032582(2677G>T/A). The rs1128503 (1236C)—rs2032582 (2677G)—rs1045642 (3435C) (C-G-C) haplotype is frequently observed in most populations [53]. This haplotype has been linked to altered P-gp function in several studies, but also these reports have been inconclusive and most found no correlation with therapeutic response [53, 54, 57]. Since rs2032582 was not available for analysis in our study (given this SNP is tri-allelic), we analyzed the combination of rs1045642 and rs1128503. In antidepressant users, we found an increased fall risk in the homozygote variant allele carriers (AA-AA) compared to the non-variant (GG-GG) genotype carriers. Although it is difficult to draw conclusions from this, due to the incomplete haplotype, it could be a first sign that determining the full haplotype could be relevant in antidepressant-related fall risk.

Our study has several limitations. First, in the harmonized cohort despite the large number of participants with genetic data the number of participants in the different genotypes was relatively low due to relatively uncommon genetic variants. Second, is the cross-sectional character of the study. Although cross-sectional studies are usually vulnerable to recall bias, no prospective data on falls was available for all included cohorts. Third, in this cross-sectional study ascertainment of antidepressant use was done at study visits, not at time of the fall. Thus, antidepressant usage during the fall incident might have differed. This may have led to misclassification and as such dilution of the effect. Therefore, it is necessary to validate our findings in a study with a prospective study design. Fourth, because we analyzed prevalent use of antidepressants, confounding by contraindication or depletion of susceptibles might have biased the associations towards the null. Fifth, as the majority of the study population was of Caucasian ethnicity, our results cannot be extrapolated to other ethnic groups. Sixth, for both *CYP2D6* and *CYP2C19* no data was available on the duplicate active alleles leading to the ultrarapid metabolizer phenotype, as duplication of genes could not be detected by the genome arrays used in our cohorts. However, duplication of CYP2D6 occur in about 1–2 percent in Caucasian Dutch [27, 58]. Seventh, CYP2D6 is highly polymorphic with over 100 known allelic variants [59], however, we were limited to two of the known variants. Eight, multiple metabolic pathways are involved in the metabolism of most drugs and for example combinatorial phenotypes based on both *CYP2D6* and *CYP2C19* genes, should be assessed in future studies. Eight, different polymorphisms can affect the pharmacokinetics and pharmacodynamics of each antidepressant differently and this distinction could not be made in our analyses.

### Clinical implications and future perspectives

There is an increasing need to better personalize antidepressant therapy to optimize treatment effects and prevent adverse events like falling. Our current study has no direct clinical implications, since our findings are novel and need to be validated with a prospective design. In general, clinical guidelines have already been developed for different *CYP2D6* and *CYP2C19* phenotypes, and recommendations are provided regarding dosing of the antidepressant and drug choice. For example, for amitriptyline and nortriptyline users a 50% dose reduction is strongly advised in case of *CYP2D6* and *CYP2C19* PM phenotype [20, 21]. Concerning future research, investigating the pharmacogenomics effects during the first few weeks after initiating antidepressant therapy would be of interest, as fall risk appears to be highest in this time frame [60]. Furthermore, studies on blood concentration levels in relation to fall risk are needed to confirm our hypothesized mechanism.

## Conclusion

This is the first study to show that genetic variation appears to modify the association between antidepressant use and fall risk. Polymorphisms in *CYP2D6*, *CYP2C19*, *CYP2C9* and *ABCB1* modified the association between antidepressant use and fall risk, although the findings were not consistent across the studies. Before firm conclusions can be made, future research with a prospective design is needed. Nonetheless, our results are a first sign that possibly implementation of pharmacogenetic applications in antidepressant (de)prescribing in falls prevention will be of clinical value in the future.

## Supporting information

S1 Appendix Cohort description. (DOCX)

S2 Appendix Covariate assessment. (DOCX)

S1 Table Chromosome positions. Chromosome position is based on assembly GRCh37.p13. R^2^ allele correlation was calculated with a help of LDpair tool (LDlink | An Interactive Web Tool for Exploring Linkage Disequilibrium in Population Groups (nih.gov)).
(DOCX)

S2 Table Details regarding covariate assessment in different cohorts and harmonization algorithms. (DOCX)

S3 Table Phenotypes of SNPs available in harmonized dataset. WT = wild type; HeZ = Heterozygous for variant allele; HoZ = Homozygous for variant allele; EM = Extensive Metabolizer; IM = Intermediate Metabolizer; PM = Poor Metabolizer.
(DOCX)

S4 Table Baseline characteristics of antidepressant users and non-antidepressant users. ^a^ mean (SD), ^b^ presented as n (%), ^c^ presented as median (IQR), ^d^ mean z-scores (SD), ^e^ data available in LASA and ERGO-5; HADS = Hospital Anxiety and Depression scale; MMSE = Mini-Mental State Examination; GFR = Glomerular filtration rate according to Cockcroft and Gault formula. * statistically significant at p-value <0.05.
(DOCX)

S5 Table Allele frequencies and distribution test according to Hardy-Weinberg equilibrium. Analysis of cases with complete medication, fall and genetic data. Values are presented as N. Deviation of allele frequencies from Hardy-Weinberg equilibrium was assessed using a Chi-Square test. * If p<0.05, SNP is not consistent with Hardy Weinberg Equilibrium.
(DOCX)

S6 Table Allele frequencies of rs1799853 and distribution test according to Hardy-Weinberg equilibrium in each cohort. Analysis of cases with complete medication, fall and genetic data. Values are presented as N. Deviation of allele frequencies from Hardy-Weinberg equilibrium was assessed using a Chi-Square test. * If p<0.05, SNP is not consistent with Hardy Weinberg Equilibrium.
(DOCX)

S7 Table Association between antidepressant use and fall risk, stratified for CYP2D6*41 genotype and CYP2D*41/*4 phenotype. Data is presented in odds ratio and 95% confidence interval. Model 1 was adjusted for age and gender. N = number of participants per genotype (total includes also participants not using antidepressants). EM = Extensive metabolizer; IM = Intermediate metabolizer; PM = Poor metabolizer. ^#^ rs28371725 (*41) & rs3892097 (*4) combined. ^$^ users of the following antidepressants were included in the exposed category: amitriptyline, nortriptyline, clomipramine, imipramine, paroxetine, fluvoxamine, citalopram, sertraline, doxepin, duloxetine, mirtazapine, venlafaxine, trazodone. * statistically significant at p<0.05.
(DOCX)

S8 Table Association between antidepressant use and fall risk, stratified for CYP2C9*3 genotype. Data is presented in odds ratio and 95% confidence interval. Model 1 was adjusted for age and gender. N = number of participants per genotype (total includes also participants not using antidepressants). ^#^ users of the following antidepressants were included in the exposed category: fluoxetine (and psycholeptics), escitalopram, amitriptyline (and psycholeptics). *statistically significant at p<0.05.
(DOCX)

S9 Table Association between antidepressant use and fall risk, stratified for ABCB1 (rs1045642) and rs1045642/rs1128503 haplotype. Data is presented in odds ratio and 95% confidence interval. Model 1 was adjusted for age and gender. N = number of participants per genotype (total includes also participants not using antidepressants). *statistically significant at p<0.05.
(DOCX)

S10 Table Association between candidate SNPs and fall risk in SSRI users in UKBB. ^a^ EA Effect allele, ^b^ NEA Non-effect allele, ^c^ EAF Effect allele frequency, ^d^ normal function allele, ^e^ deficient function allele. *statistically significant at p<0.05.
(DOCX)

S11 Table Association between candidate SNPs and fall risk in TCA users in UKBB. ^a^ EA Effect allele, ^b^ NEA Non-effect allele, ^c^ EAF Effect allele frequency, ^d^ normal function allele, ^e^ deficient function allele. *statistically significant at p<0.05.
(DOCX)
