# Supplementary material for: Candidate genetic variants and antidepressant-related fall risk in middle-aged and older adults
Source: PLoS One. 2022 Apr 14;17(4):e0266590. doi: 10.1371/journal.pone.0266590 (PMC9009709; doi:10.1371/journal.pone.0266590)
Supplement: S1 Appendix — (DOCX) [file pone.0266590.s001.docx]

**S1 Appendix** - **Cohort description**

The Rotterdam study, or Erasmus Rotterdam Gezondheid Onderzoek (ERGO), is a population-based prospective cohort study aiming to assess the determinants and prognosis of chronic diseases in older persons. ERGO-5 wave was incorporated in the harmonized cohort. This wave is the fifth examination of the first cohort, the third examination of the second cohort and the second examination of the third ERGO cohort. These examination cycles took place between 2009 and 2013. Since 2008, the cohort comprised 14,926 participants >50 years from a suburb of Rotterdam [1, 2].

B-PROOF is a large multicenter, randomized, double-blind, placebo-controlled trial of 2,919 participants, aged 65 years or older, with elevated homocysteine levels and recruited between 2008 and 2011. The primary aim was to assess whether the addition of vitamin B12 and folic acid to vitamin D therapy had added value in the prevention of osteoporotic fractures. Previous results indicated no effect of the intervention on fall-related outcomes. Therefore, the trial was applicable for harmonization with the other cohorts [3, 4].

LASA is a prospective cohort study that started in 1992 and included older persons between 55 and 85 years old. A random sample was drawn from the population of several municipalities of the Netherlands and was stratified for age and gender. LASA was initiated to study the determinants and consequences of physical, cognitive, emotional, and social functioning in relation to ageing^.^ The harmonized cohort compromised data of 2,545 subjects from LASA wave C, which took place between 1995-1996 and from which 1,509 respondents participated in the fall follow-up. LASA wave 3B formed by a new inclusion of 1,023 subjects between 2012-2013 [5, 6].

The UKBB is a large population-based longitudinal study of approximately half-million adult (aged 40-69 years) participants living in the United Kingdom (UK), recruited from 22 centers across the UK in 2006-2010. The GWAS for drug-related falls included data from approximately 34,000 antidepressant users [7]. More detailed descriptions of the studies are provided elsewhere [1-7].

**References**

1. Hofman A, Grobbee DE, de Jong PT, van den Ouweland FA. Determinants of disease and disability in the elderly: the Rotterdam Elderly Study. European journal of epidemiology. 1991;7(4):403-22. Epub 1991/07/01. doi: 10.1007/bf00145007. PubMed PMID: 1833235.

2. Ikram MA, Brusselle G, Ghanbari M, Goedegebure A, Ikram MK, Kavousi M, et al. Objectives, design and main findings until 2020 from the Rotterdam Study. European journal of epidemiology. 2020;35(5):483-517. Epub 2020/05/06. doi: 10.1007/s10654-020-00640-5. PubMed PMID: 32367290; PubMed Central PMCID: PMCPMC7250962.

3. Swart KM, Ham AC, van Wijngaarden JP, Enneman AW, van Dijk SC, Sohl E, et al. A Randomized Controlled Trial to Examine the Effect of 2-Year Vitamin B12 and Folic Acid Supplementation on Physical Performance, Strength, and Falling: Additional Findings from the B-PROOF Study. Calcified tissue international. 2016;98(1):18-27. Epub 2015/09/29. doi: 10.1007/s00223-015-0059-5. PubMed PMID: 26412463; PubMed Central PMCID: PMCPMC4703626.

4. van Wijngaarden JP, Dhonukshe-Rutten RA, van Schoor NM, van der Velde N, Swart KM, Enneman AW, et al. Rationale and design of the B-PROOF study, a randomized controlled trial on the effect of supplemental intake of vitamin B12 and folic acid on fracture incidence. BMC geriatrics. 2011;11:80. Epub 2011/12/06. doi: 10.1186/1471-2318-11-80. PubMed PMID: 22136481; PubMed Central PMCID: PMCPMC3266639.

5. Hoogendijk EO, Deeg DJH, de Breij S, Klokgieters SS, Kok AAL, Stringa N, et al. The Longitudinal Aging Study Amsterdam: cohort update 2019 and additional data collections. European journal of epidemiology. 2020;35(1):61-74. Epub 2019/07/28. doi: 10.1007/s10654-019-00541-2. PubMed PMID: 31346890; PubMed Central PMCID: PMCPMC7058575.

6. Huisman M, Poppelaars J, van der Horst M, Beekman AT, Brug J, van Tilburg TG, et al. Cohort profile: the Longitudinal Aging Study Amsterdam. International journal of epidemiology. 2011;40(4):868-76. Epub 2011/01/11. doi: 10.1093/ije/dyq219. PubMed PMID: 21216744.

7. Sudlow C, Gallacher J, Allen N, Beral V, Burton P, Danesh J, et al. UK biobank: an open access resource for identifying the causes of a wide range of complex diseases of middle and old age. PLoS medicine. 2015;12(3):e1001779. Epub 2015/04/01. doi: 10.1371/journal.pmed.1001779. PubMed PMID: 25826379; PubMed Central PMCID: PMCPMC4380465.
