# Supplementary material for: Candidate genetic variants and antidepressant-related fall risk in middle-aged and older adults
Source: PLoS One. 2022 Apr 14;17(4):e0266590. doi: 10.1371/journal.pone.0266590 (PMC9009709; doi:10.1371/journal.pone.0266590)
Supplement: S2 Appendix — (DOCX) [file pone.0266590.s002.docx]

**S2 Appendix – Covariate assessment**

Baseline characteristics were collected by questionnaires and by performing measurements at study visits in all cohorts. Living situation was categorized into community-dwelling or institutionalized. The highest level of completed education was harmonized in accordance with the International Standard Classification of Education (ISCED 2011) mappings [1]. Three categories of educational level were created: low educated (ISCED level 0, 1 and 2), average educated (ISCED level 3 and 4) and high educated (ISCED level 5 through 8). Use of alcohol was categorized into five groups: non-drinkers, drinking less than once a month, drinking 1-3 times a month, drinking 1-4 days a week and drinking almost daily. Smoking habit was categorized into non-smoker or current smoker. From weight and height, the body mass index (BMI in kg/m2) was calculated. Systolic and diastolic blood pressures were measured at baseline. For the variable hypotension the lowest measured values were used, where a cut-off value of ≤120mHg systolic and or ≤ 70mmHg diastolic was considered as hypotension [2-4]. Use of co-medication was obtained in the different cohorts as described in the methods. For this study, number of medication was defined and the use of benzodiazepines, antipsychotics and opioids was of particular interest.

Depressive symptoms in the LASA 3B and C wave and ERGO cohort were initially measured by the Center for Epidemiological Studies Depression Scale (CES-D) and in B-PROOF by the geriatric depression scale (GDS). After harmonization, depressive symptoms were expressed in z-scores. Cognitive functioning was measured in all cohorts through the Mini-Mental State Examination (MMSE). Anxiety was measured in LASA 3B and C wave and ERGO with the HADS anxiety scale (HADSA). In B-PROOF, no anxiety scale was present. For the presence of pain, the general occurrence of pain in the last six months was asked in the ERGO, while in the LASA cohort, current pain was reported. In B-PROOF, Euroqol item 4 was used (range 1-3: pain or other complaints). After harmonization, pain variable was categorized into a dichotomous variable, the presence or absence of pain. Serum creatinine levels were used to calculate age-adjusted estimate of the glomerular filtration rate according to the Cockcroft and Gault formula. Diabetes was based on self-reporting information. Information about dizziness was only available in the LASA and ERGO-5 cohorts and was harmonized into regular presence of dizziness.

In all cohorts, grip strength was measured by a dynamometer, but with different types (LASA-C and B-PROOF: Takei TKK 5001, Takei Scientific Instruments Co. Ltd., Tokyo, Japan, LASA 3B JAMAR 5030J1 Hydraulic Hand Dynamometer). Therefore Z-scores were computed. Also, for gait speed z-scores were computed for each cohort, since the cohorts used different measurements (3-meter walking tests in B-PROOF and LASA-cohorts and in the Rotterdam study gait assessment with a 5.79-m-long electronic walkway (GAITRite Platinum;CIR systems Inc.,Sparta, NJ,USA). Balance was measured using the tandem stance test in all cohorts and was harmonized into unable or able to balance for more than 10 seconds. For all cohorts, information about the use of walking aid during the walking test was available and this was categorized into a dichotomous variable.

**References**

1. International Standard Classification of Education (ISCED). <http://uis.unesco.org/en/isced-mappings2011>.

2. Klein D, Nagel G, Kleiner A, Ulmer H, Rehberger B, Concin H, et al. Blood pressure and falls in community-dwelling people aged 60 years and older in the VHM&PP cohort. BMC geriatrics. 2013;13:50. Epub 2013/05/23. doi: 10.1186/1471-2318-13-50. PubMed PMID: 23692779; PubMed Central PMCID: PMCPMC3663706.

3. Muller M, Smulders YM, de Leeuw PW, Stehouwer CD. Treatment of hypertension in the oldest old: a critical role for frailty? Hypertension (Dallas, Tex : 1979). 2014;63(3):433-41. Epub 2013/12/11. doi: 10.1161/hypertensionaha.113.00911. PubMed PMID: 24324042.

4. Bromfield SG, Ngameni CA, Colantonio LD, Bowling CB, Shimbo D, Reynolds K, et al. Blood Pressure, Antihypertensive Polypharmacy, Frailty, and Risk for Serious Fall Injuries Among Older Treated Adults With Hypertension. Hypertension (Dallas, Tex : 1979). 2017;70(2):259-66. Epub 2017/06/28. doi: 10.1161/hypertensionaha.116.09390. PubMed PMID: 28652459; PubMed Central PMCID: PMCPMC5667360.
