# Supplementary material for: Candidate genetic variants and antidepressant-related fall risk in middle-aged and older adults
Source: PLoS One. 2022 Apr 14;17(4):e0266590. doi: 10.1371/journal.pone.0266590 (PMC9009709; doi:10.1371/journal.pone.0266590)
Supplement: S1 Table — Chromosome position is based on assembly GRCh37.p13. R2 allele correlation was calculated with a help of LDpair tool (LDlink | An Interactive Web Tool for Exploring Linkage Disequilibrium in Population Groups (nih.gov)). (DOCX) [file pone.0266590.s003.docx]

**S1 Table – Chromosome positions**

| Gene | rs-number | Chromosome | Chromosome position | R^2^ allele correlation* |
| --- | --- | --- | --- | --- |
| CYP1A2 | rs762551 | 15 | 75041917 |  |
| CYP3A4 | rs35599367 | 7 | 99366316 |  |
| CYP3A5 | rs77646 | 7 | 99270539 |  |
| CYP2C9 | rs1799853 | 10 | 96702047 | R**^2^**: 0.0111 for rs1799853 and rs1057910 |
| CYP2C9 | rs1057910 | 10 | 96741053 |  |
| CYP2D6 | rs28371725 | 22 | 42523805 | R**^2^**: 0.0235 for rs28371725 and rs3892097 |
| CYP2D6 | rs3892097 | 22 | 42524947 |  |
| CYP2C19 | rs4244285 | 10 | 96541616 | R**^2^**: 0.0489 for rs4244285 and rs12248560 |
| CYP2C19 | rs12248560 | 10 | 96521657 |  |
| ABCB1 | rs1045642 | 7 | 87138645 | R**^2^**: 0.4357 for rs1045642 and rs1228503 |
| ABCB1 | Rs1128503 | 7 | 87179601 |  |
| Chromosome position is based on assembly GRCh37.p13.  R^2^ allele correlation was calculated with a help of LDpair tool (LDlink \| An Interactive Web Tool for Exploring Linkage  Disequilibrium in Population Groups (nih.gov)) | | | | |
