# Supplementary material for: Candidate genetic variants and antidepressant-related fall risk in middle-aged and older adults
Source: PLoS One. 2022 Apr 14;17(4):e0266590. doi: 10.1371/journal.pone.0266590 (PMC9009709; doi:10.1371/journal.pone.0266590)
Supplement: S2 Table — (DOCX) [file pone.0266590.s004.docx]

**S2 Table - Details regarding covariate assessment in different cohorts and harmonization algorithms.**

| Variable | Categories | Harmonization algorithm/Details | Cohorts available |
| --- | --- | --- | --- |
| Gender | Male/female |  | All cohorts |
| Age | N/A |  | All cohorts |
| Living status | Community dwelling/institutionalized | LASA:  -living independently categorized as community-dwelling  -answer options residential home, nursing home, hospital, psychiatric hospital and monastery categorized as institutionalized  B-PROOF:  -living independently categorized as community-dwelling  -answer options assisted living, service flat and home for the elderly categorized as institutionalized  Rotterdam study:  -living independently categorized as community-dwelling  - answer options service flat, residential care home and nursing home categorized as institutionalized | All cohorts |
| Education | Low/average/high | Categorized according ISCED 2011 mapping (low/average/high)  LASA:  -Education level attained  -answer options elementary not completed (5yrs), elementary (6yrs), lower vocational (9yrs) and general intermediate (10yrs) categorized as low  intermediate vocational (11yrs), general secondary (12 yrs) categorized as average-higher vocational (15yrs), college (16 yrs), university (18yrs) categorized as high  B-PROOF:  - highest level of education completed  -answer options: primary school (5yrs), lower education (6yrs), MULO/ULO/MAVO (9yrs), secondary education (10yrs), MMS/HBS/Lyceum/Atheneum?Gymnasium to 3^rd^ year (included) (11yrs) categorized as low  -MMS/HBS/Lyceum/Atheneum/Gymnasium completed (12yrs) categorized as average  -higher education (15yrs), university or college, until examination (16yrs), university or college, fully completed (18yrs) categorized as high  Rotterdam study:  -Highest level of education  -answer options: primary education (5yrs), lower vocational/intermediate general education (5-9yrs) categorized as low  -intermediate vocational education OR general secondary education (10-14yrs) categorized as average  -higher vocational education (15-18yrs) categorized as high | All cohorts |
| Body Mass index | N/A | Calculated from measured height and weight | All cohorts |
| Alcohol | -non-drinkers  -drinking less than once month  -drinking one to three times a month  -drinking one to four days a week  -drinking almost daily | Based on self-report:  LASA:  How many days per week do you drink alcohol (past year)?  Answer options categorized following ways:  -every day and 5-6 days/week  categorized as drinking almost daily  -1-2 days/wk and 3-4 days/wk categorized as drinking one to four days a week  - drinking one to three times a month categorized as drinking one to three times a month  - less than once in a month categorized as drinking less than once month  -not drinking categorized as non-drinkers  B-PROOF:  Did you drink alcohol last year?  Answer options categorized following ways:  -every day and 5-6 days/week  categorized as drinking almost daily  -1-2 days/wk and 3-4 days/wk categorized as drinking one to four days a week  -drinking one to three times a month categorized as drinking one to three times a month  -less than once in a month categorized as drinking less than once month  -not drinking categorized as non-drinkers  Rotterdam study:  How often did you use alcohol in the past year?  Answer options categorized following ways:  -4 times or more/wk as drinking almost daily  -2-3 days/wk categorized as drinking one to four days a week  -drinking one to four times a month categorized as drinking one to three times a month  -less than once in a month categorized as drinking less than once month  -not drinking categorized as non-drinkers | All cohorts |
| Smoking | Yes/no | Based on self-report  LASA:  -Do you smoke? Did you smoke before?  B-PROOF  -Do you smoke cigarettes, cigars, other tobacco? (never/now/past)  -Do you smoke cigarettes (yes/no)  -Number of cigarettes a day  -Do you smoke cigars (yes/no)  -Number of cigars  Rotterdam study  -do you smoke cigarettes?  -do you smoke cigars?  -do you smoke a pipe? | All cohorts |
| Depressive symptoms | N/A | Z-score depressive symptoms was created.  Following questionnaires were assessed:  LASA:  -20-item Center for Epidemiologic Studies Depression Scale (CES-D)  B-PROOF:  -Geriatric Depression Scale (GDS)  Rotterdam study:  -20-item Center for Epidemiologic Studies Depression Scale (CES-D) | All cohorts |
| Anxiety | N/A | HADS-Anxiety score was used | Not available in B-PROOF. |
| MMSE Score | N/A |  | All cohorts |
| Dizziness | Dizziness/no Dizziness | Based on self-report  LASA:  -regularly dizzy (yes/no)  Rotterdam study:  -Are you ever dizzy?  -Answer options no and almost never categorized into no dizziness and option sometimes and yes, all the time into dizziness. | Not available in B-PROOF |
| Pain | Pain/no pain | Based on self-report  Following questionnaires were assessed:  LASA:  -pain at present: Low/No pain was categorized as having no pain, answer options 6 or higher were categorized as having pain  B-PROOF:  -Euroqol item 4: range 1-3: pain or other complaints  -Answer options some and severe were categorized as pain  Rotterdam study:  -In the past 6 months, did you experience any pain  -options yes daily or weekly or a few times/monthly were categorized as pain | All cohorts |
| Handgrip strength (z-score) | N/A | Z-score of grip strength was created.  LASA:  -Grip strength dynamometer (wave C: Takei TKK 5001, Takei Scientific Instruments Co. Ltd., Tokyo, Japan. Wave 3B: JAMAR 5030J1 Hydraulic Hand Dynamometer)  -Maximum of two attempts with each hand  B-PROOF:  -strain-gauged dynamometer (Takei, TKK 5401, Takei Scientific Instruments Co. Ltd., Japan  -Maximum of two attempts with each hand    Rotterdam study:  -Fabrication Enterprises hydraulic hand dynamometer  -Maximum of three attempts | All cohorts |
| Balance | Unable/able to balance more than 10 seconds | Unable/able to balance more than 10 seconds was created based on following tests:  LASA:  -Tandem stance (up to 10 sec in C and up to 30 sec in 3B)  B-PROOF:  -Tandem stand test  Rotterdam study:  -Participants were categorized to the group “able” if they could stand on one feet or on tandem position >10 seconds | All cohorts |
| Walking aid use | walking aid used during walking test test/no walking aid | Walking aid used during walking test | All cohorts |
| Gait speed (z-score) | N/A | Z-score of gait speed was created.  LASA:  -walk test 2x 3meter walk and turn; as quickly as possible  B-PROOF:  -Timed walking test: walk 3m, turn around and walk back. As quickly as possible.  Rotterdam study:  -Gait assessment with 5.79m long electronic walkway with pressure sensors; Centimeters per second was scored. | All cohorts |
| Hypotension | Hypotension/no hypotension | A cut-off value of ≤120mHg systolic and or ≤ 70mmHg diastolic was considered as hypotension  Blood pressure was measured following way:  LASA:  -Wave 3B: Blood pressure measured in duplicate in 3B on the upper arm while sitting using an automatic Omron device (left arm, if not possible right)  -lowest of two measurements  Wave C: blood pressure was measured once in sitting position, in lying position and in standing position.  -lowest of these measurements  B-PROOF:  -Blood pressure is measured in duplicate using an Omron M1 plus blood pressure device  -lowest of two measurements  Rotterdam study:  -Blood pressure was measured at the right brachial artery with the participant in sitting position.  - lowest of two measurements | All cohorts |
| eGFR | N/A | Calculated from creatinine (Cockcroft and Gault formula) | All cohorts |
| Number of medication | N/A | -Variable was created using the number of described medication  -Supplements were removed from the number of medications | All cohorts |
