# Supplementary material for: Candidate genetic variants and antidepressant-related fall risk in middle-aged and older adults
Source: PLoS One. 2022 Apr 14;17(4):e0266590. doi: 10.1371/journal.pone.0266590 (PMC9009709; doi:10.1371/journal.pone.0266590)
Supplement: S3 Table — WT = wild type; HeZ = Heterozygous for variant allele; HoZ = Homozygous for variant allele; EM = Extensive Metabolizer; IM = Intermediate Metabolizer; PM = Poor Metabolizer. (DOCX) [file pone.0266590.s005.docx]

**S3 Table – Phenotypes of SNPs available in harmonized dataset**

| Gene | Combined SNPs | EM | IM | PM |
| --- | --- | --- | --- | --- |
| CYP2D6  [1, 2] | *4  *41 | *4 WT, *41 WT  *41 HeZ *4WT  *41 WT, *4 HZ  *41 Hoz *4WT | *41 HeZ + 4* Hez  *41 HoZ *4 HeZ | *4 HoZ + Any *41 |
|  |  | **Non-variant allele carriers** | **Variant allele carriers** |  |
| CYP2C9 [3] | *2  *3 | *1/*1 | *1/*2  *1/*3  *2/*3  *2/*2  *3/*3 |  |
|  |  | **Reference** | **Heterozygous** | **Homozygous** |
| ABCB1 [4] | Rs1045642  Rs1128503 | GG + GG | GA + GA | AA + AA |
| WT= wild type; HeZ=Heterozygous for variant allele; HoZ= Homozygous for variant allele  EM=Extensive Metabolizer; IM=Intermediate Metabolizer; PM= Poor Metabolizer | | | | |

**References**

1. Hicks JK, Bishop JR, Sangkuhl K, Muller DJ, Ji Y, Leckband SG, et al. Clinical Pharmacogenetics Implementation Consortium (CPIC) Guideline for CYP2D6 and CYP2C19 Genotypes and Dosing of Selective Serotonin Reuptake Inhibitors. Clinical pharmacology and therapeutics. 2015;98(2):127-34. Epub 2015/05/15. doi: 10.1002/cpt.147. PubMed PMID: 25974703; PubMed Central PMCID: PMCPMC4512908.

2. Hicks JK, Sangkuhl K, Swen JJ, Ellingrod VL, Muller DJ, Shimoda K, et al. Clinical pharmacogenetics implementation consortium guideline (CPIC) for CYP2D6 and CYP2C19 genotypes and dosing of tricyclic antidepressants: 2016 update. Clinical pharmacology and therapeutics. 2017;102(1):37-44. Epub 2016/12/21. doi: 10.1002/cpt.597. PubMed PMID: 27997040; PubMed Central PMCID: PMCPMC5478479.

3. Ham AC, Ziere G, Broer L, Swart KM, Enneman AW, van Dijk SC, et al. CYP2C9 Genotypes Modify Benzodiazepine-Related Fall Risk: Original Results From Three Studies With Meta-Analysis. Journal of the American Medical Directors Association. 2017;18(1):88.e1-.e15. Epub 2016/11/28. doi: 10.1016/j.jamda.2016.09.021. PubMed PMID: 27889507.

4. Genvigir FD, Salgado PC, Felipe CR, Luo EY, Alves C, Cerda A, et al. Influence of the CYP3A4/5 genetic score and ABCB1 polymorphisms on tacrolimus exposure and renal function in Brazilian kidney transplant patients. Pharmacogenet Genomics. 2016;26(10):462-72. Epub 2016/07/20. doi: 10.1097/fpc.0000000000000237. PubMed PMID: 27434656.
