# Supplementary material for: Candidate genetic variants and antidepressant-related fall risk in middle-aged and older adults
Source: PLoS One. 2022 Apr 14;17(4):e0266590. doi: 10.1371/journal.pone.0266590 (PMC9009709; doi:10.1371/journal.pone.0266590)
Supplement: S4 Table — a mean (SD), b presented as n (%), c presented as median (IQR), d mean z-scores (SD), e data available in LASA and ERGO-5; HADS = Hospital Anxiety and Depression scale; MMSE = Mini-Mental State Examination; GFR = Glomerular filtration rate according to Cockcroft and Gault formula. * statistically significant at p-value <0.05. (DOCX) [file pone.0266590.s006.docx]

**S4 Table - Baseline characteristic of antidepressant users and non-antidepressant users**

|  | Variable available | Antidepressant use  (n= 700) | Non antidepressant use (n= 11,065) | P-value |
| --- | --- | --- | --- | --- |
| Cohort ^b^  B-PROOF  ERGO-5  LASA C  LASA 3B | 11,765 | 115 (16.4)  481 (68.7)  40 (5.7)  64 (9.1) | 2,144 (19.4)  6,635 (60)  1164 (13.2)  822 (7.4) | <0.001* |
| Age (years) ^b^  50-59 years  60-69 years  70-79 years  80+ | 11,765 | 115 (16.4)  265 (37.9)  228 (32.6)  92 (13.1) | 1,507 (13.6)  3,731 (33.7)  3,824 (34.6)  2,003 (18.7) | 0.001* |
| Gender ^b^  male  female | 11,765 | 203 (29)  497 (71) | 5,096 (46.1)  5,969 (53.9) | <0.001* |
| Community-dwelling (yes) ^b^ | 10,608 | 572 (88.8) | 9,218 (92.5) | 0.001* |
| Education^b^  Low  Average   High | 11,690 | 402 (57.8)  162 (23.3)  132 (19) | 6,123 (55.7)  2,490 (22.6)  2,381 (21.7) | 0.244 |
| Body Mass Index (kg/m^2^) ^a^ | 10,833 | 27.9 (4.96) | 27.3 (4.28) | .006 |
| Alcohol use ^b^  Non-drinker  Rarely drinks  Low risk (≤ 14 drinks per week)  Risky (15-28 drinks per week)  High risk (>28 drinks per week) | 11,736 | 193 (27.6)  91 (13)  322 (46.1)  68 (9.7)  25 (3.6) | 1,726 (15.6)  1,435 (13)  5,840 (52.9)  1,631 (14.8)  405 (3.7) | <0.001* |
| Smoking (yes) ^b^ | 11,755 | 131 (18.7) | 1,438 (13) | <0.001* |
| Depressive symptoms  (z-score) ^d^ | 11,290 | 0.70 (1.43) | -0.05 (0.94) | <0.001* |
| Anxiety (HADS-A)  ^ce^ | 9,311 | 4 (1-7) | 2 (0-4) | <0.001* |
| MMSE score ^c^ | 5,037 | 28 (26-29) | 28 (26-29) | 0.493 |
| Dizziness (yes) ^be^ | 7,194 | 161 (35.7) | 1,252 (18.6) | <0.001* |
| Pain (yes) ^b^ | 7,303 | 230 (61.2) | 3,029 (43.7) | <0.001* |
| Handgrip strength (z-score) ^d^ | 10,599 | -0.29 (0.88) | 0.03 (0.99) | <0.001* |
| Balance (unable) ^b^ | 8,215 | 75 (17.4) | 1,415 (18.2) | 0.667 |
| Walking aid use (yes)  ^b^ | 8,268 | 13 (3) | 130 (1.7) | 0.035 |
| Gait speed (z-score)^d^ | 8,890 | -0.25 (0.99) | 0.02 (0.98) | <0.001* |
| Hypotension (yes)^b^ | 10,644 | 191 (31.9) | 2,727 (27.1) | 0.012 |
| eGFR (ml min^-1^  1.73m²)^c^ | 10,209 | 77.4 (63.6-96.2) | 75.6 (61.6-92) | 0.001* |
| Number of medications  ^a^ | 11,764 | 4.76 (2.35) | 2.79 (2.45) | <0.001* |
| Benzodiazepine use (yes) ^b^ | 11,765 | 167 (23.9) | 771 (7.0) | <0.001* |
| Opioid use (yes) ^b^ | 11,765 | 75 (10.7) | 379 (3.4) | <0.001* |
| Antipsychotic use (yes) ^b^ | 11,765 | 41 (5.9) | 52 (0.5) | <0.001* |
| ^a^ mean (SD), ^b^ presented as n (%), ^c^ presented as median (IQR), ^d^ mean z-scores (SD)^, e^ data available in LASA and ERGO-5; HADS= Hospital Anxiety and Depression scale; MMSE=Mini-Mental State Examination; GFR=Glomerular filtration rate according to Cockcroft and Gault formula  * statistically significant at p-value <0.05 | | | | |
