# Supplementary material for: Candidate genetic variants and antidepressant-related fall risk in middle-aged and older adults
Source: PLoS One. 2022 Apr 14;17(4):e0266590. doi: 10.1371/journal.pone.0266590 (PMC9009709; doi:10.1371/journal.pone.0266590)
Supplement: S5 Table — Analysis of cases with complete medication, fall and genetic data. Values are presented as N. Deviation of allele frequencies from Hardy-Weinberg equilibrium was assessed using a Chi-Square test. * If p<0.05, SNP is not consistent with Hardy Weinberg Equilibrium. (DOCX) [file pone.0266590.s007.docx]

**S5 Table- Allele frequencies and distribution test according to Hardy-Weinberg equilibrium.**

|  | All participants  N= 9,335 |
| --- | --- |
| ABCB1: rs1045642 |  |
| AA  GA  GG  X^2^  p-value | 2,655  4,610  2,070  0.662  0.415 |
| rs1128503 |  |
| AA  GA  GG  X^2^  p-value | 1,694  4,621  3,020  1.014  0.313 |
| CYP1A2: rs762551 |  |
| AA  CA  CC  X^2^  p-value | 4,968  3,700  667  0.38  0.54 |
| CYP2D6: rs28371725 |  |
| CC  CT  TT  X^2^  p-value | 7768  1488  79  0.687  0.406 |
| rs3892097 |  |
| CC  CT  TT  X^2^  p-value | 5,795  3,083  457  3.21  0.07 |
| CYP3A4: rs35599367 |  |
| AA  AG  GG  X^2^  p-value | 31  1,082  8,222  0.53  0.47 |
| CYP2C9: rs1799853 |  |
| CC  CT  TT  X^2^  p-value | 7,174  1,992  169  5.0  0.025* |
| rs1057910 |  |
| AA  CA  CC  X^2^  p-value | 8,172  1,126  37  0.07  0.787 |
| CYP2C19: rs4244285 |  |
| AA  AG  GG  X^2^  p-value | 222  2,397  6,716  0.219  0.639 |
| rs12248560 |  |
| CC  TC  TT  X^2^  p-value | 5,656  3,203  476  0.667  0.414 |
| CYP3A5: rs776746 |  |
| CC  TC  TT  X^2^  p-value | 8110  1190  35  1.528  0.216 |
| Analysis of cases with complete medication, fall and genetic data.  Values are presented as N. Deviation of allele frequencies from Hardy-Weinberg equilibrium was assessed using a Chi-Square test.  * If p<0.05, SNP is not consistent with Hardy Weinberg Equilibrium | |
