# Supplementary material for: Candidate genetic variants and antidepressant-related fall risk in middle-aged and older adults
Source: PLoS One. 2022 Apr 14;17(4):e0266590. doi: 10.1371/journal.pone.0266590 (PMC9009709; doi:10.1371/journal.pone.0266590)
Supplement: S6 Table — Analysis of cases with complete medication, fall and genetic data. Values are presented as N. Deviation of allele frequencies from Hardy-Weinberg equilibrium was assessed using a Chi-Square test. * If p<0.05, SNP is not consistent with Hardy Weinberg Equilibrium. (DOCX) [file pone.0266590.s008.docx]

**S6 Table - allele frequencies of rs1799853 and distribution test according to Hardy-Weinberg equilibrium in each cohort.**

| CYP2C9 | B-PROOF | ERGO | LASA C | LASA 3B |
| --- | --- | --- | --- | --- |
| Rs1799853 |  |  |  |  |
| CC | 1,532 | 4,545 | 663 | 434 |
| CT | 423 | 1,265 | 187 | 117 |
| TT | 40 | 95 | 27 | 7 |
| X^2^ | 2.841 | 0.418 | 8.852 | 0.079 |
| p-value | 0.09 | 0.517 | 0.0029* | 0.778 |
| Analysis of cases with complete medication, fall and genetic data.  Values are presented as N. Deviation of allele frequencies from Hardy-Weinberg equilibrium was assessed using a  Chi-Square test.  * If p<0.05, SNP is not consistent with Hardy Weinberg Equilibrium | | | | |
