# Supplementary material for: Candidate genetic variants and antidepressant-related fall risk in middle-aged and older adults
Source: PLoS One. 2022 Apr 14;17(4):e0266590. doi: 10.1371/journal.pone.0266590 (PMC9009709; doi:10.1371/journal.pone.0266590)
Supplement: S7 Table — Data is presented in odds ratio and 95% confidence interval. Model 1 was adjusted for age and gender. N = number of participants per genotype (total includes also participants not using antidepressants). EM = Extensive metabolizer; IM = Intermediate metabolizer; PM = Poor metabolizer. # rs28371725 (*41) & rs3892097 (*4) combined. $ users of the following antidepressants were included in the exposed category: amitriptyline, nortriptyline, clomipramine, imipramine, paroxetine, fluvoxamine, citalopram, sertraline, doxepin, duloxetine, mirtazapine, venlafaxine, trazodone. * statistically significant at p<0.05. (DOCX) [file pone.0266590.s009.docx]

**S7 Table - Association between antidepressant use and fall risk, stratified for CYP2D6*41 genotype and CYP2D6*41/*4 phenotype**

|  | All antidepressant users | | | Substrate specific antidepressant users ^$^ | | |
| --- | --- | --- | --- | --- | --- | --- |
| CYP2D6 *41 | **N** | **Model 1** | **P-value** | **N** | **Model 1** | **P-value** |
| CC | 7768 | 1.86 (1.53-2.26) | <0.001* | 7768 | 1.90 (1.49-2.41) | <0.001* |
| TC | 1488 | 0.92 (0.53-1.61) | 0.779 | 1488 | 0.99 (0.50-1.93) | 0.965 |
| TT | 79 | 0.95 (0.16-5.52) | 0.950 | 79 | - | - |
| Any variant allele carriers  (TC and TT) | 1567 | 0.93 (0.55-1.58) | 0.799 | 1567 | 0.88 (0.45-1.71) | 0.703 |
| CYP2D6 combined phenotype ^#^ | **N** | **Model 1** | **P-value** | **N** | **Model 1** | **P-value** |
| EM | 8522 | 1.73 (1.43-2.09) | <0.001* | 8522 | 1.74 (1.38-2.20) | <0.001* |
| IM | 356 | 0.55 (0.15-1.98) | 0.361 | 356 | 0.60 (0.13-2.89) | 0.528 |
| PM | 457 | 2.17 (1.02-4.61) | 0.044* | 457 | 2.19 (0.85-5.62) | 0.103 |
| IM and PM combined | 810 | 1.43 (0.77-2.66) | 0.256 | 810 | 1.47 (0.68-3.17) | 0.324 |
| Data is presented in odds ratio and 95% confidence interval. Model 1 was adjusted for age and gender.  N = number of participants per genotype (total includes also participants not using antidepressants).  EM= Extensive metabolizer; IM= Intermediate metabolizer; PM= Poor metabolizer  ^#^ rs28371725 (*41) & rs3892097 (*4) combined  ^$^ users of the following antidepressants were included in the exposed category: amitriptyline, nortriptyline, clomipramine, imipramine, paroxetine, fluvoxamine, citalopram, sertraline, doxepin, duloxetine, mirtazapine, venlafaxine, trazodone  * statistically significant at p<0.05 | | | | | | |
