# Supplementary material for: Candidate genetic variants and antidepressant-related fall risk in middle-aged and older adults
Source: PLoS One. 2022 Apr 14;17(4):e0266590. doi: 10.1371/journal.pone.0266590 (PMC9009709; doi:10.1371/journal.pone.0266590)
Supplement: S8 Table — Data is presented in odds ratio and 95% confidence interval. Model 1 was adjusted for age and gender. N = number of participants per genotype (total includes also participants not using antidepressants). # users of the following antidepressants were included in the exposed category: fluoxetine (and psycholeptics), escitalopram, amitriptyline (and psycholeptics). *statistically significant at p<0.05. (DOCX) [file pone.0266590.s010.docx]

**S8 Table - Association between antidepressant use and fall risk, stratified for CYP2C9*3 genotype**

|  | All antidepressant users | | | Substrate specific antidepressant users ^#^ | | |
| --- | --- | --- | --- | --- | --- | --- |
| CYP2C9 *3 | **N** | **Model 1** | **P-value** | **N** | **Model 1** | **P-value** |
| AA | 8172 | 1.56 (1.28-1.89) | <0.001* | 8172 | 1.57 (1.06-2.33) | 0.024* |
| CA | 1126 | 2.98 (1.82-4.88) | <0.001* | 1126 | 2.39 (0.91-6.28) | 0.078 |
| CC | 37 | - | - | 37 | - | - |
| Any variant allele carriers (CA & CC) | 1163 | 3.02 (1.84-4.93) | <0.001* | 1163 | 2.41 (0.92-6.35) | 0.074 |
| Data is presented in odds ratio and 95% confidence interval. Model 1 was adjusted for age and gender. N = number of participants per genotype (total includes also participants not using antidepressants).  ^#^ users of the following antidepressants were included in the exposed category: fluoxetine (and psycholeptics), escitalopram, amitriptyline (and psycholeptics)  *statistically significant at p<0.05 | | | | | | |
