# Supplementary material for: Candidate genetic variants and antidepressant-related fall risk in middle-aged and older adults
Source: PLoS One. 2022 Apr 14;17(4):e0266590. doi: 10.1371/journal.pone.0266590 (PMC9009709; doi:10.1371/journal.pone.0266590)
Supplement: S9 Table — Data is presented in odds ratio and 95% confidence interval. Model 1 was adjusted for age and gender. N = number of participants per genotype (total includes also participants not using antidepressants). *statistically significant at p<0.05. (DOCX) [file pone.0266590.s011.docx]

**S9 Table - Association between antidepressant use and fall risk, stratified for ABCB1 (rs1045642) and rs1045642/rs1128503 haplotype.**

|  | All antidepressant users | | |
| --- | --- | --- | --- |
| rs1045642 | **N** | **Model 1** | **P-value** |
| GG | 2070 | 1.17 (0.78-1.75) | 0.439 |
| AG | 4610 | 1.92 (1.48 – 2.48) | <0.001* |
| AA | 2655 | 1.83 (1.31-2.54) | <0.001* |
| Variant allele carriers  (GA & AA) | 7265 | 1.89 (1.54-2.31) | <0.001* |
| rs1045642/rs11285003 |  |  |  |
| GGGG | 1769 | 1.20 (0.78-1.84) | 0.409 |
| GAGA | 3303 | 1.79 (1.32-2.44) | <0.001* |
| AAAA | 1443 | 2.19 (1.44-3.33) | <0.001* |
| Variant allele carriers  (GAGA & AAAA) | 4746 | 1.92 (1.50-2.46) | <0.001* |
| Data is presented in odds ratio and 95% confidence interval. Model 1 was adjusted for age and gender. N = number of participants per genotype (total includes also participants not using antidepressants).  *statistically significant at p<0.05 | | | |
