# Supplementary material for: Candidate genetic variants and antidepressant-related fall risk in middle-aged and older adults
Source: PLoS One. 2022 Apr 14;17(4):e0266590. doi: 10.1371/journal.pone.0266590 (PMC9009709; doi:10.1371/journal.pone.0266590)
Supplement: S10 Table — a EA Effect allele, b NEA Non-effect allele, c EAF Effect allele frequency, d normal function allele, e deficient function allele. *statistically significant at p<0.05. (DOCX) [file pone.0266590.s012.docx]

**S10 Table - Association between candidate SNPs and fall risk in SSRI users in UKBB**

| **Gene** | **SNP** | **EA^a^** | **NEA^b^** | **EAF^c^** | **OR (95 % CI)** | **P-value** |
| --- | --- | --- | --- | --- | --- | --- |
| **ABCB1** | rs1045642 | A | G | 0.54 | 1.01 (0.97 -1.05) | 0.628 |
|  | rs1128503 | A | G | 0.44 | 0.99 (0.95- 1.04) | 0.712 |
| **CYP3A4** | rs35599367 (*22) | G | A | 0.95 | 1.02 (0.93- 1.13) | 0.626 |
| **CYP3A5** | rs776746 | C | T | 0.93 | 1.01 (0.93-1.10) | 0.750 |
| **CYP2C9** | rs1057910 (*3) | A | C | 0.94 | 0.98 (0.90-1.07) | 0.630 |
|  | rs1799853 (*2) | C ^d^ | T ^e^ | 0.87 | 1.08 (1.01 - 1.15) | 0.018* |
| **CYP2C19** | rs4244285(*2) | G | A | 0.85 | 0.96 (0.91- 1.02) | 0.185 |
|  | rs12248560 (*17) | C | T | 0.79 | 0.99 (0.9- 1.04) | 0.668 |
| **CYP1A2** | rs762551(*1F) | C | A | 0.27 | NA |  |
| **CYP2D6** | rs28371725(*41) | T | C | 0.10 | 1.07 (1.00-1.15) | 0.051 |
|  | rs3892097 (*4) | T | C | 0.20 | 0.99 (0.94-1.04) | 0.624 |
| ^a^ EA= Effect allele, **^b^** NEA =Non-effect allele, **^c^** EAF= Effect allele frequency, ^d^ normal function allele, ^e^ deficient function allele  *statistically significant at p<0.05 | | | | | | |
