# Supplementary material for: Candidate genetic variants and antidepressant-related fall risk in middle-aged and older adults
Source: PLoS One. 2022 Apr 14;17(4):e0266590. doi: 10.1371/journal.pone.0266590 (PMC9009709; doi:10.1371/journal.pone.0266590)
Supplement: S11 Table — a EA Effect allele, b NEA Non-effect allele, c EAF Effect allele frequency, d normal function allele, e deficient function allele. *statistically significant at p<0.05. (DOCX) [file pone.0266590.s013.docx]

**S11 Table - Association between candidate SNPs and fall risk in TCA users in UKBB**

| **Gene** | **SNP** | **EA^a^** | **NEA^b^** | **EAF^c^** | **OR (95 % CI)** | **P-value** |
| --- | --- | --- | --- | --- | --- | --- |
| **ABCB1** | rs1045642 | A | G | 0.54 | 1.03 (0.98-1.09) | 0.228 |
|  | rs1128503 | A | G | 0.44 | 1.05 (0.99-1.11) | 0.086 |
| **CYP3A4** | rs35599367 (*22) | G | A | 0.95 | 1.04 (0.93-1.18) | 0.492 |
| **CYP3A5** | rs776746 | C | T | 0.93 | 1.08 (0.97-1.20) | 0.139 |
| **CYP2C9** | rs1057910 (*3) | A | C | 0.94 | 0.98 (0.87-1.09) | 0.660 |
|  | rs1799853 (*2) | C | T | 0.86 | 0.97 (0.90-1.04) | 0.392 |
| **CYP2C19** | rs4244285(*2) | G | A | 0.85 | 0.95 (0.88-1.02) | 0.165 |
|  | rs12248560 (*17) | C | T | 0.79 | 1.02 (0.96-1.09) | 0.476 |
| **CYP1A2** | rs762551(*1F) | C | A | 0.28 | 0.99 (0.94-1.05) | 0.808 |
| **CYP2D6** | rs28371725(*41) | T ^e^ | C ^d^ | 0.10 | 1.12 (1.02-1.22) | 0.014* |
|  | rs3892097 (*4) | T | C | 0.20 | 0.96 (0.89-1.02) | 0.173 |
| ^a^ EA= Effect allele, **^b^** NEA =Non-effect allele, **^c^** EAF= Effect allele frequency, ^d^ normal function allele, ^e^ deficient function allele  *statistically significant at p<0.05 | | | | | | |
