# Supplementary material for: Candidate genetic variants and antidepressant-related fall risk in middle-aged and older adults
Source: PLoS One. 2022 Apr 14;17(4):e0266590. doi: 10.1371/journal.pone.0266590 (PMC9009709; doi:10.1371/journal.pone.0266590)
Supplement: Attachment — Submitted filename: Response to Reviewers.docx [file pone.0266590.s014.docx]

Anouschka Pronk

Amsterdam UMC, location AMC

Meibergdreef 9; 1105 AZ Amsterdam, The Netherlands

+31 205665991

[a.c.pronk@amsterdamumc.nl](mailto:a.c.pronk@amsterdamumc.nl)

Francesc Calafell
Academic Editor
PLOS ONE

February 23th, 2022

Dear Francesc Calafell,

Subject: submission of revised paper "Candidate genetic variants and antidepressant-related fall risk in middle-aged and older adults "/ PONE-D-21-37547

Thank you for the opportunity to revise our manuscript: Candidate genetic variants and antidepressant-related fall risk in middle-aged and older adults

We describe our changes to the manuscript on the subsequent pages. Our responses to the reviewers and comments appear in italics. Changed sections are written in blue and track changes are visible.

All the changes in the revised manuscript have been done with track of changes. We have revised the manuscript according to reviewer’s critique.

We look forward to hearing from you.

Sincerely,

Anouschka Pronk, on behalf of all co-authors.

**Reviewer #1:**

**This paper analyses the possible relation between some candidate pharmacogenes and falls in older antidepressant users. Overall, the methodology, results, and conclusions seem sound.
Although I think that the paper is mostly suitable for publication, I do find, however, that it might also benefit from some improvements:**

*We thank the reviewer for the thorough review of the manuscript.*

1. **Gene CYP3A5 is mentioned in Table 5 and Suppl. T1, but is missing from the text.**

*Thank you for noticing this, we have added CYP3A5 to the text of the methods section. Please see the following (lines: 96-100):*

*The following non-rare variants (minor allele frequency (MAF) >1% in Caucasian population) that could be genotyped from genome-wide arrays were identified [27, 28]: CYP1A2: rs762551 (*1F); CYP3A4: rs35599367 (*22); CYP3A5: rs776746 (*3); CYP2C9:rs1799853 (*2); rs1057910 (*3); CYP2D6: rs28371725 (*41); rs3892097 (*4); CYP2C19: rs4244285 (*2); rs12248560 (*17); ABCB1: rs1045642; rs1128503.*

**2. 6 cohorts are mentioned in the Methods, but only 5 are named (LASA, B-PROOF, ERGO, Irish LSA, AFE Ulm), and only 3 are explained in Suppl. 1 (ERGO, B-PROOF, LASA).**

*We have corrected this to five studies. In the harmonized cohort, there are six cohorts from five studies, since LASA has two cohorts from different time periods, but for the clarity, we corrected the sentence. Please see the following (line 108):*

*This is a harmonized cohort of five European cohorts (the Longitudinal Aging Study Amsterdam (LASA), the B-vitamins for the Prevention Of Osteoporotic Fractures (B-PROOF), the Rotterdam Study (or Erasmus Rotterdam Gezondheid Onderzoek (ERGO), the Irish Longitudinal Study on Ageing and the Activity and Function in the Elderly in Ulm study)*

*In the supporting information we describe in more detail the included cohorts that contain genetic data, in which we were able to test our research question. To clarify this better we corrected the sentence. Please see the following (line 111 -115):*

*Since only the LASA, B-PROOF and ERGO cohort have genetic data available, this subset was included in the current study.. More detailed description of these cohorts can be found in the supporting information (S1 Appendix). Subsequently, we leveraged data from a genome-wide association study (GWAS) for drug-related falls performed in UKBB.*

**3. Falls are considered in the previous 12 months, but no time is considered for antidepressant use - I'm not sure whether this is accounted for in the limitations of the paper.**

*We have adjusted the sentence to indicate this limitation more clearly. Please see the following (line 402-405):*

*Third, in this cross-sectional study ascertainment of antidepressant use was done at study visits, not at time of the fall. Thus, the antidepressant usage during the fall incident might have differed. This may have led to misclassification and as such dilution of the effect. Therefore, it is necessary to validate our findings in a study with a prospective study design.*

**4. It is not clear why some antidepressant categories have been analyzed separately but others (monoamine oxidase inhibitors, i.e. N06AF and N06AG) are analyzed together with "other antidepressants" (N06AX)**

*Thank you for pointing this out. N06AF, N06AG categories were combined with N06AX as there needed to be sufficient amount of users per variable. We determined that the group size needed to be >1% of the participants. The following sentence has been added to the manuscript (line: 136-138)*

*N06AF, N06AG categories were combined with N06AX (into category: ‘other antidepressants’) as there needed to be sufficient amount of users per variable. The antidepressant variables needed to be used by >1% of the participants.*

**5. It is not clear why some possible covariates were not analyzed in Model 2 (e.g. dizzines, balance...)**

*We thank the reviewer mentioning this. We did not include these variables in model 2, since we considered these variables as possible mediators. They could be in the pathway between antidepressant use and falls. We have clarified this in the methods section by adding the following sentence (line 153-154):*

*Possible mediators between antidepressant use and falls (dizziness, balance, hypotension, gait speed) were not tested as covariates.*

**6. it is not clear why the cut-off of multicollinearity is set at 0.4 and not another value**

*Various cut-offs (0.4-0.85) have been applied in the literature. We applied the more restrictive cut-off off 0.4 to be sure that the mulitcollinearity problem is not present in our analysis. We have added this to the methods section with adding a reference (Dormann 2013). Please see the following (line 189-191):*

*Various cut-offs (0.4-0.85) have been applied in the literature [44]. We applied the more restrictive cut-off of ≥0.4 for the coefficient to be sure that the multicollinearity problem is not present in our analyses*

**7. I am not sure whether the results of the supplementary tables regarding the associations between antidepressant use and fall risk stratified for genotypes are correct (e.g. Number of participants per genotype in group "All antidepressant users" being equal to group "Substrate specific antidepressant users", and generally being superior to the reported total number of Antidepressant users (N = 700)).**

*In the analyses in which we stratified per genotype, the totals are higher since they also include the non-users of antidepressants. We have clarified this in the relevant tables in the appendix. Please see the following: S7 Table, S8 Table and S9 Table.*

*N= number of participants per genotype (total includes also participants not using antidepressants).*

**8.It is not clear why the "alcohol use" covariate has been limited to groups with no use and high use**

*We added the other categories of alcohol use to the baseline characteristics table. Please see the following: S4 Table*

*Alcohol use*

*Non-drinker*

*Rarely drinks*

*Low risk (≤ 14 drinks per week)*

*High risk (> 28 drinks per week)*

**9. Some limitations of the study are not stated: trans people appear to be excluded from the analysis, all participants seem to have a BMI > 27 (overweight)**

*Thank you for commenting on these issues.*

*Unfortunately, in the different cohorts we used, at the time of the inclusion, participants were only classified as male or female. Transgenders were not intentionally excluded.*

*We noticed that we have erroneously placed standard error instead of standard deviation for the BMI in the table (S4 Table). We have adjusted this and also for the variables to which the same applied: depressive symptoms and handgrip strength. Furthermore, we are aware that the mean BMI is relatively high. Unfortunately this is in accordance with current BMI values of the current western population.*

*If the editor whishes we can add the points about transgenders and BMI to the limitation section.*

**10. Terms "gender" and "sex" seem to be used interchangeably or not properly explained (e.g. "gender" in data table but "models adjusted by sex")**

*Thank you for pointing this out. Since baseline characteristics were not scored based on genetics we consider the term “gender” as the right term. We have corrected this throughout the manuscript. Please see the following changes: line 147, line 178, line 180, Table 1, S1 Appendix, S2 Table.*

**Editorial note**

**1.Please ensure that your manuscript meets PLOS ONE's style requirements, including those for file naming. The PLOS ONE style templates can be found at**

*We have checked the PLOS ONE’s style requirements and ensured that the manuscripts meets the requirements.*

**2.** **We note that the grant information you provided in the ‘Funding Information’ and ‘Financial Disclosure’ sections do not match.**

*Thank you for pointing this out. The text listed below applies to both funding information and financial disclosure. In the funding information section we added all relevant grant numbers.*

This work was supported by the Clementine Brigitta Maria Dalderup fund (project numbers 20713 and 7303, receiver N. van der Velde), which is an Amsterdam University fund (<https://www.auf.nl/en/about-the-fund/about.html>). The funder had no role in study design, data collection and analysis, decision to publish, or preparation of the manuscript.

The initial B-PROOF study has received funding so far by the Netherlands Organization for Health Research and Development (ZonMw, Grant 6130.0031), The Hague; unrestricted grant from NZO (Dutch Dairy Association), Zoetermeer; Orthica, Almere; Netherlands Consortium Healthy Ageing (NCHA) Leiden/Rotterdam; Ministry of Economic Affairs, Agriculture and Innovation (project KB-15-004-003), The Hague; Wageningen University, Wageningen; VUmc, Amsterdam; Erasmus Medical Center, Rotterdam.

The Longitudinal Aging Study Amsterdam (LASA) is largely supported by a grant from the Netherlands Ministry of Health, Welfare and Sports, Directorate of Long-Term Care. The data collection in 2012–2013 was financially supported by the Netherlands Organization for Scientific Research (NWO) in the framework of the project “New Cohorts of young old in the 21st century” (File Number 480-10-014).

The Rotterdam Study is supported by the Erasmus MC University Medical Center and Erasmus University Rotterdam; The Netherlands Organization for Scientific Research (NWO); The Netherlands Organization for Health Research and Development (ZonMw); the Research Institute for Diseases in the Elderly (RIDE); The Netherlands Genomics Initiative (NGI); the Ministry of Education, Culture and Science; the Ministry of Health, Welfare and Sports; the European Commission (DG XII); and the Municipality of Rotterdam.

The UK Biobank was established by the Welcome Trust medical charity, Medical Research Council, Department of Health, Scottish Government and the Northwest Regional Development Agency. It has also received funding from the Welsh Government, British Heart Foundation, Cancer Research UK and Diabetes UK. UK Biobank is supported by the National Health Service (NHS). UK Biobank is open to bona fide researchers anywhere in the world, including those funded by academia and industry. The medical research project is a non-profit charity which has received core funding of around £133 Million. Core funding continues to be received from the Welcome Trust, the MRC, and more recently, from Cancer Research UK and NIHR. UK Biobank has received additional funding for: genotyping of all 500,000 participants (from the Department of Health, Medical Research Council, British Heart Foundation)

The sponsors have no role in the design or implementation of the study, data collection, data management, data analysis, data interpretation, or in the preparation, review, or approval of the manuscript.

**3. We note that you have indicated that data from this study are available upon request.**

*Unfortunately because of ethical and legal restrictions we are not allowed to share our data publicly, but the data is available on request.. The following text can be added to the section of data availability.*

*Because of restrictions based on privacy regulations and informed consent of the participants, data cannot be made freely available in a public repository.*

*Data from the LASA database are available for use for specific research questions provided that an agreement is made up (*[*https://www.lasavu.nl/data/availability_data/availability_data.htm*](https://www.lasavu.nl/data/availability_data/availability_data.htm)*). Rotterdam study data can be obtained upon request. Requests should be directed towards the management team of the Rotterdam Study (*[*secretariat.epi@erasmusmc.nl*](mailto:secretariat.epi@erasmusmc.nl)*), which has a protocol for approving data request. B-PROOF data can be obtained upon request. Requests should be directed towards the principal investigators of the studies, who have a protocol for approving data requests. Data from the UK Biobank are available on application to the UK Biobank Access Committee. The Access Committee may be contacted by email (*[*access@ukbiobank.ac.uk*](mailto:access@ukbiobank.ac.uk)*) or through the UK Biobank web page (*[*http://www.ukbiobank.ac.uk*](http://www.ukbiobank.ac.uk)*)*

**4. Please review your reference list to ensure that it is complete and correct. If you have cited papers that have been retracted, please include the rationale for doing so in the manuscript text, or remove these references and replace them with relevant current references**.

*We checked all our references for possible retraction.*
